# Perceived Knowledge, Attitudes, and Barriers Toward the Adoption of Telemedicine Services in the Kingdom of Saudi Arabia: Cross-Sectional Study

**DOI:** 10.2196/46446

**Published:** 2023-07-27

**Authors:** Saeed Baradwan, Mohammed Al-Hanawi

**Affiliations:** 1 Department of Obstetrics and Gynecology King Faisal Specialist Hospital and Research Center Jeddah Saudi Arabia; 2 Department of Health Services and Hospital Administration King Abdulaziz University Jeddah Saudi Arabia; 3 Health Economics Research Group King Abdulaziz University Jeddah Saudi Arabia

**Keywords:** telemedicine, telehealth, attitude, knowledge, barrier, Saudi Arabia, mobile phone

## Abstract

**Background:**

The adoption of telemedicine has facilitated the opportunity to offer the best health care services. Nevertheless, there is a mismatch between the presence of telemedicine programs in the Kingdom of Saudi Arabia and poor acceptance by the end-user patients.

**Objective:**

This study aimed to gain a holistic understanding of the knowledge, attitudes, and barriers of the end-user patients (ie, research participants) toward the utility of telemedicine services in the Kingdom of Saudi Arabia.

**Methods:**

A cross-sectional, survey-based study was conducted from June 1 to July 31, 2022, in the Kingdom of Saudi Arabia. The questionnaire was developed based on a literature review and was examined for validity and reliability. Knowledge questions used a yes-or-no format, whereas attitude and barrier questions used a 5-point Likert-scale format. Data were reported descriptively and analyzed using SPSS (IBM Corp) software. To evaluate the differences in mean scores and identify sociodemographic factors associated with knowledge and attitudes toward the adoption of telemedicine, the data were analyzed using univariate and multivariable regression analyses, respectively.

**Results:**

Overall, 1024 participants participated in the survey. The percentages of participants who attended a telemedicine service before, during, and after COVID-19 were 49.61% (508/1024), 61.91% (634/1024), and 50.1% (513/1024), respectively. The mean score for knowledge was 3.52 (SD 1.486; range 0-5), reflecting a high level of knowledge. The mean score for attitudes was 37.08 (SD 8.526; range 11-55), reflecting optimistic (positive) attitudes. Regarding barriers, the participants voiced some concerns regarding patient and physician resistance and attributed some cultural and technology-related limitations as potential hindrances to the full adoption of telemedicine services. The place of residence (rural vs nonrural) had a significant impact on the scores of knowledge, attitudes, and barriers, whereas gender had no significant impact. The multivariable regression analysis showed that several sociodemographic factors significantly correlated with knowledge and attitudes toward the adoption of telemedicine services.

**Conclusions:**

The participants showed good knowledge and positive attitudes toward telemedicine services. The perceived barriers were in line with the published literature. This research calls for the need to strengthen the positive attitudes and rectify the barriers, so that the utility of telemedicine services can be maximized in the community.

## Introduction

### Background

Swift developments in information and communications technology have occurred over the past few decades and culminated in substantial benefits to the health sector of high-income countries [1]. Various forms of information technologies, for example, mobile health, eHealth, telehealth, and telemedicine, have been depicted to considerably decrease health care expenses and human-made medical errors [2,3]. Telemedicine, in particular, is a pivotal contributor to health care accessibility in resource-limited countries, such as the Kingdom of Saudi Arabia [4]. According to the World Health Organization, telemedicine is understood as “the delivery of healthcare services, where distance is a critical factor, by all the healthcare professionals using information and communications technology for the exchange of valid information for the diagnosis, treatment, and prevention of disease, research and evaluation.” Telemedicine can also be recognized as the capacity to offer outstanding health care delivery, irrespective of time and space, through the utility of telecommunication technologies [5].

**Figure 1:**
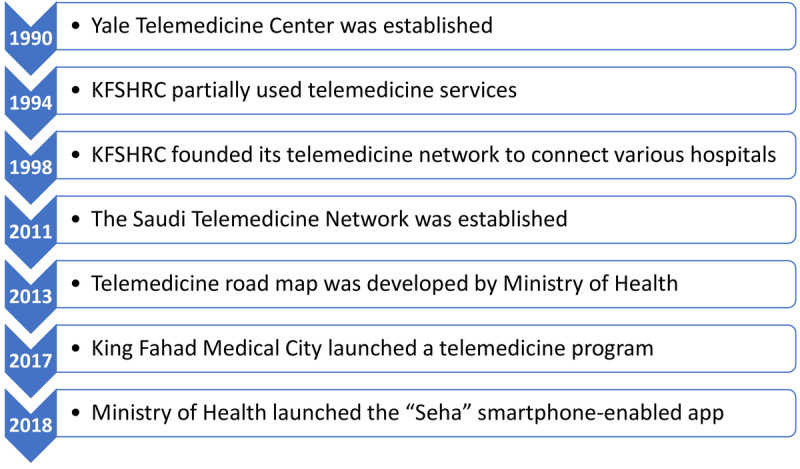
Summary of the timeline of telemedicine history and progress in Saudi Arabia. KFSHRC: King Faisal Specialist Hospital & Research Center.

In high-income countries, particularly the European countries such as the United Kingdom, Sweden, and Germany, the adoption of telemedicine has facilitated the opportunity to offer the best health care services [6]. Such wide-ranging benefits of telemedicine include cost-effectiveness, reduced frequency of medical errors, facilitation of care coordination, better diagnosis and treatment, improved disease prevention, and reduction of unnecessary waiting times [7,8]. Unfortunately, in contrast, resource-limited countries, particularly Middle Eastern countries, including the Kingdom of Saudi Arabia, are making slow growth in embracing relevant IT advances to promote the wide implementation and utility of national telemedicine programs [6]. Indeed, although the Kingdom of Saudi Arabia has established several telemedicine programs in the early 1990s (Figure 1), the progress by policy makers on one side and the rate of use by providers (health care personnel) and acceptors (patients) on the other side are not in line with the expectations [9]. Generally, the reasons for poor acceptance of telemedicine programs are multifactorial [9].

Health care personnel and patients are the 2 primary end users of telemedicine services. Several studies have investigated the perceived attitudes and barriers toward the utility of telemedicine services among health care professionals [10-12]. Conversely, few studies exist in place about the perceptions, attitudes, and knowledge of patients toward the utility and barriers of telemedicine services in the Kingdom of Saudi Arabia [13]. This area of research has been recently reported as a deficient topic in the literature according to a systematic thematic review of eHealth services in the Gulf Cooperation Council countries [13,14].

### Objectives

Therefore, the main aim of this research was to gain a holistic understanding of the perceptions and barriers of the end-user participants toward the utility of telemedicine in the Kingdom of Saudi Arabia. Our research is noteworthy from a conceptual perspective because it focuses on telemedicine, which is a very important topic relevant to health care. The importance of telemedicine is demonstrated by the wide array of benefits that are heavily documented in the literature [7,8]. This research aimed to fill out this fissure in the literature by contributing valuable data that can be beneficially exploited by policy makers to devise strategic plans to maximize the utility of telemedicine and its acceptability by participants in the Kingdom of Saudi Arabia. To the best of our knowledge, the detailed impact of sociodemographics on the likelihood to adopt telemedicine services has not been extensively investigated. These data will provide crucial knowledge to formulate structured plans to address the key sociodemographic determinants of adopting telemedicine services by participants.

## Methods

### Study Design and Sampling

This study used a cross-sectional (June 1 to July 31, 2022), survey-based research design. It was conducted in a web-based, anonymous, and optional (voluntary) fashion.

On the basis of the latest census data from the Kingdom of Saudi Arabia (mid-2021), the population of Saudi Arabia was estimated to be 34,110,821 according to the General Authority for Statistics in the Kingdom of Saudi Arabia. A sample size calculator was used to calculate the required sample size for the study. On the basis of a 5% margin of error, a 95% CI, a 50% response distribution, and a population size of 34,110,821, the calculated sample size was 385 participants. To account for unforeseen circumstances, a desired population of at least 1000 participants was targeted, and the plan was to recruit as many responses as possible. The targeted participants included all those without any sociodemographic restrictions, including, but not limited to, age, gender, race, ethnicity, income status, nationality, religion, and familiarity with technology. Participants with incomplete data were excluded.

Two types of nonprobability sampling techniques were used. First, convenience sampling was used in which participants were selected for inclusion in the sample because they were very easy for the researcher to be accessed, as these participants received a direct WhatsApp link to the questionnaire. Second, a simplified snowball sampling technique was used in which some participants were specifically asked to send the WhatsApp link of the questionnaire to their contacts. The WhatsApp link to the questionnaire was distributed widely through various social media platforms, such as WhatsApp groups, Facebook, Twitter, and Instagram. Notably, the study was based on an anonymous survey, and participation was voluntary. By agreeing to participate in the survey, participants provided implied consent, and then they were directed to complete the survey. There were no incentives to participate in this study.

### Measurement Tool and Data Collection

The study questionnaire was created based on a literature review [15-17], and some new questions deemed important by the authors were also added to the questionnaire. The questionnaire was written in English and then translated into Arabic. The Arabic version was used to administer the questionnaire. Afterward, the questionnaire was examined for 2 elements to ensure high quality and authentic reporting of the findings. These elements included study validity and reliability [18]. Validity was examined through pilot-testing by distributing the survey to 20 participants to ensure proper interpretation of questions, and some questions were modified accordingly before producing the final questionnaire. In contrast, reliability was tested using the Cronbach α coefficient test, which was used to measure the extent of internal consistency among the tested items. A value of >.70, indicating an acceptable internal reliability of the data, was sought; otherwise, the question was omitted [19].

The questionnaire was divided into five main parts: (1) sociodemographics (n=14 questions), (2) the proportion of participants who had used telemedicine services before, (3) knowledge about telemedicine services (n=5 questions), (4) perceived attitudes toward the adoption of telemedicine services by participants (n=11 questions), and (5) perceived barriers toward the adoption of telemedicine services by participants (n=6 questions). The knowledge questions were scored according to a yes-or-no format. The questions on perceived attitudes and barriers were scored on a 5-point Likert scale as follows: 1=strongly disagree, 2=disagree, 3=neutral, 4=agree, and 5=strongly agree.

The survey was conducted over the web using Google Forms. The WhatsApp link to the survey was shared via various social media platforms, such as WhatsApp, Facebook, Twitter, and Instagram. The data were collected and tabulated using Microsoft Excel (Microsoft Corporation) for analysis. The data were collected from June 1 to July 31, 2022.

### Independent Variables

For sociodemographic variables, the age variable was divided into 5 categories: 18 to 29 years (reference category), 30 to 39 years, 40 to 49 years, 50 to 59 years, and ≥60 years. Gender was coded 1 for men and 0 for women. Marital status was divided into 4 categories: single, married, widowed, and divorced. For analysis purposes, marital status was captured as binary, with a value of 1 for married status and 0 for otherwise. Education level was divided into 5 categories: primary school completed or below (reference category), secondary or high school completed, diploma before university completed, college or university degree completed, and postgraduate degree completed. Nationality was divided into 2 categories: non-Saudi (reference group) and Saudi. The number of family members was divided into 2 categories: ≤5 (reference group) and ≥6. Job status was divided into 6 categories: unemployed (reference group), governmental, nongovernmental, self-employed, and retired. Monthly income was divided into 8 categories: <Saudi riyal (SAR) 3000 (reference category; a currency exchange rate of SAR 1=US $0.27 is applicable); SAR 3000 to <SAR 5000; SAR 5000 to <SAR 7000; SAR 7000 to <SAR 10,000; SAR 10,000 to <SAR 15,000; SAR 15,000 to <SAR 20,000; SAR 20,000 to <SAR 30,000; and ≥SAR 30,000. The place of residence was divided into 13 regions: Riyadh (reference category), Makkah, Eastern Region, Asir, Jizan, Almadinah, Alqassim, Tabuk, Najran, Hai’l, Aljouf, Albaha, and Northern Borders Region. Other sociodemographic variables included living in rural area, coexistence of chronic illness, availability of transportation vehicle, availability of smartphone, and availability of social media account; all of which were captured as binary with a value of 0 for *no* (reference group) and 1 for *yes*.

### Dependent Variables

For the knowledge questions (n=5 questions), scores of 0 and 1 were allocated to *no* and *yes* answers, respectively (range 0-5). The questions on perceived attitudes (n=11 questions) and barriers (n=6 questions) were scored on a 5-point Likert scale, with scores ranging from 11-55 and 6-30, respectively. Scores were computed by averaging answers of the participants to all questions within the category. The average 5-point Likert scale responses were presented as means (SDs; range: minimum-maximum). Responses of strongly disagree, disagree, neutral, agree, and strongly agree corresponded to numerical values of 1, 2, 3, 4, and 5, respectively, during the statistical analysis. Higher scores correlated with better knowledge and positive attitudes.

### Analysis Methods

Data were analyzed using SPSS (version 24.0; IBM Corp). For each knowledge question, we deemed a response of >50% as a good knowledge. For each attitude statement, we deemed overall mean scores of <3, 3, and >3 as negative, neutral, and positive attitudes, respectively. Categorical data were presented as numbers and percentages. A 2-tailed chi-square test of independence was used for the univariate analysis (relationships) of categorical data. A 2-tailed Student *t* test or 1-way ANOVA was used for univariate analysis of numerical data. Detailed analyses of knowledge and attitudes toward telemedicine services were conducted according to 2 specific sociodemographic variables: gender and rural area. A multivariable regression analysis was performed to identify sociodemographic variables related to knowledge and attitudes, as done previously [20]. The scores were logged for all variables and interpreted using ordinary least squares. Higher scores indicated better knowledge and positive attitudes. The sum of the responses for each participant was calculated using a Microsoft Excel sheet, and the logged values were then computed. The logged value of the summation of responses was regressed against all the dummy variables. The corresponding coefficient (β) and SE were tabulated for the questions on knowledge and attitudes. Statistical significance was determined as *P*<.05 (α error of 5%).

### Ethics Approval

The study protocol was approved by the institutional review board and Ethical Committee at King Faisal Specialist Hospital and Research Center, Jeddah, Saudi Arabia (2022-17).

## Results

### Characteristics of the Surveyed Population

A total of 1024 individuals participated in the survey. Table 1 summarizes the baseline characteristics of the surveyed population. Most participants were married (518/1024, 50.59%), had completed college or university education (313/1024, 30.57%), were Saudi citizens (769/1024, 75.1%), and had jobs (653/1024, 63.77%). The surveyed individuals differed in age, salary, and number of family members. The 3 regions with the highest response rates were Makkah (279/1024, 27.25%), Riyadh (150/1024, 14.65%), and Eastern Region (111/1024, 10.84%). A large proportion of the surveyed participants had cars (734/1024, 71.68%), smartphones (859/1024, 83.89%), and at least 1 social media account (802/1024, 78.32%). Only 26.17% (268/1024) and 59.57% (610/1024) of the individuals reported living in rural areas and having chronic diseases, respectively. The percentages of participants who attended a telemedicine service before, during, and after COVID-19 were 49.61% (508/1024), 61.91% (634/1024), and 50.1% (513/1024), respectively. For all participants, the mean score for knowledge was 3.52 (SD 1.486; range 0-5), reflecting a high level of knowledge. The mean score for attitude was 37.08 (SD 8.526; range 11-55), reflecting optimistic (positive) attitudes.

**Table 1:** Baseline characteristics of the surveyed population (N=1024).

Demographic characteristic			Value
**Age group (years), n (%)**			
	18-29	213 (20.8)	
	30-39	309 (30.18)	
	40-49	227 (22.17)	
	50-59	166 (16.21)	
	≥60	109 (10.64)	
**Gender, n (%)**			
	Men	504 (49.22)	
	Women	520 (50.78)	
**Marital status, n (%)**			
	Single	259 (25.29)	
	Married	518 (50.59)	
	Widowed	141 (13.77)	
	Divorced	106 (10.35)	
**Education level, n (%)**			
	Primary school completed and below	125 (12.21)	
	Secondary or high school completed	270 (26.37)	
	Diploma before university completed	135 (13.18)	
	College or University degree completed	313 (30.57)	
	Postgraduate degree completed	181 (17.67)	
**Nationality, n (%)**			
	Non-Saudi	255 (24.9)	
	Saudi	769 (75.1)	
**Job, n (%)**			
	Government employee	331 (32.32)	
	Nongovernment employee	209 (20.41)	
	Self-employed	113 (11.03)	
	Student	128 (12.5)	
	Retired	117 (11.43)	
	Unemployed	126 (12.3)	
**Number of family members, n (%)**			
	≤5	708 (69.14)	
	≥6	316 (30.86)	
**Monthly household income (SAR^a^), n (%)**			
	<3000	156 (15.23)	
	3000 to <5000	101 (9.86)	
	5000 to <7000	136 (13.28)	
	7000 to <10,000	146 (14.26)	
	10,000 to <15,000	191 (18.65)	
	15,000 to <20,000	140 (13.67)	
	20,000 to <30,000	95 (9.28)	
	≥30,000	59 (5.76)	
**Place of residence, n (%)**			
	Riyadh Region	150 (14.65)	
	Makkah Region	279 (27.25)	
	Eastern Region	111 (10.84)	
	Asir Region	63 (6.15)	
	Jizan Region	75 (7.32)	
	Almadinah Region	72 (7.03)	
	Alqassim Region	57 (5.57)	
	Tabuk Region	38 (3.71)	
	Haíl Region	36 (3.51)	
	Najran Region	28 (2.73)	
	Aljouf Region	30 (2.93)	
	Albaha Region	47 (4.59)	
	Northern Borders Region	38 (3.71)	
**Live in rural areas, n (%)**			
	No	756 (73.83)	
	Yes	268 (26.17)	
**Chronic illness, n (%)**			
	No	610 (59.57)	
	Yes	414 (40.43)	
**Availability of transportation car, n (%)**			
	No	290 (28.32)	
	Yes	734 (71.68)	
**Availability of smartphone, n (%)**			
	No	165 (16.11)	
	Yes	859 (83.89)	
**Availability of social media account, n (%)**			
	No	222 (21.68)	
	Yes	802 (78.32)	

^a^SAR: Saudi riyal. SAR 1=US $0.27.

### Summary of the Impact of Sociodemographic Factors on the Overall Knowledge and Attitudes Toward Telemedicine Services

Table 2 summarizes the differences in overall knowledge and attitudes according to the sociodemographic factors of the surveyed participants. The scores of knowledge were statistically different for all examined sociodemographic factors, except gender and having a chronic disease. In contrast, the scores of attitudes were statistically different for all examined sociodemographic factors, except gender, number of family members, salary, and having a chronic disease.

**Table 2:** Comparison of the mean scores of knowledge and attitudes according to the sociodemographic characteristics (N=1024)^a^.

Variable		Participant, n (%)	Knowledge score			Attitude score			
			Value, mean (SD)	*P* value^b^		Value, mean (SD)		*P* value^b^	
**Age group (years)**					.02				.004
	18-29	213 (20.8)	3.5 (1.507)			37.43 (8.286)			
	30-39	309 (30.18)	3.74 (1.465)			38.38 (8.45)			
	40-49	227 (22.17)	3.41 (1.397)			35.71 (8.839)			
	50-59	166 (16.21)	3.34 (1.564)			36.8 (8.173)			
	≥60	109 (10.64)	3.38 (1.514)			35.96 (8.615)			
**Gender**					.26				.67
	Men	504 (49.22)	3.46 (1.548)			36.96 (8.505)			
	Women	520 (50.78)	3.57 (1.423)			37.19 (8.553)			
**Marital status**					<.001				<.001
	Single	259 (25.29)	3.49 (1.498)			37.43 (8.086)			
	Married	518 (50.59)	3.72 (1.407)			38.26 (8.168)			
	Widowed	141 (13.77)	3.24 (1.483)			34.33 (8.696)			
	Divorced	106 (10.35)	2.96 (1.65)			34.09 (9.591)			
**Nationality**					<.001				<.001
	Non-Saudi	255 (24.9)	2.94 (1.518)			33.61 (8.006)			
	Saudi	769 (75.1)	3.71 (1.426)			38.23 (8.386)			
**Number of family members**					.04				.63
	≤5	708 (69.14)	3.58 (1.48)			37.16 (8.561)			
	≥6	316 (30.86)	3.37 (1.493)			36.88 (8.457)			
**Education**					<.001				<.001
	Primary school completed and below	125 (12.21)	2.6 (1.344)			31.91 (7.24)			
	Intermediate school completed	88 (8.6)	2.89 (1.572)			33.73 (7.648)			
	High school completed	182 (17.77)	3.73 (1.584)			38.07 (9.49)			
	Diploma before university completed	135 (13.18)	3.61 (1.511)			37.13 (9.027)			
	College or university degree completed	313 (30.57)	3.76 (1.369)			38.82 (7.562)			
	Postgraduate degree completed	181 (17.67)	3.74 (1.288)			38.22 (8.173)			
**Job**					<.001				<.001
	Government employee	331 (32.32)	3.87 (1.372)			39.36 (8.555)			
	Nongovernment employee	209 (20.41)	3.66 (1.495)			37.66 (8.91)			
	Self-employed	113 (11.03)	2.98 (1.482)			33.38 (7.824)			
	Student	128 (12.5)	3.19 (1.478)			34.95 (7.835)			
	Retired	117 (11.43)	3.28 (1.455)			35.81 (8.203)			
	Unemployed	126 (12.3)	3.37 (1.558)			36.75 (7.548)			
**Salary (SAR^c^)**					<.001				.10
	<3000	156 (15.23)	3.04 (1.632)			36.1 (8.196)			
	3000 to <5000	101 (9.86)	3.26 (1.677)			36.16 (7.91)			
	5000 to <7000	136 (13.28)	3.57 (1.443)			37.17 (8.772)			
	7000 to <10,000	146 (14.26)	3.69 (1.451)			36.8 (8.291)			
	10,000 to <15000	191 (18.65)	3.63 (1.423)			37.62 (8.943)			
	15,000 to <20,000	140 (13.67)	3.65 (1.373)			38.22 (8.531)			
	20,000 to <30,000	95 (9.28)	3.86 (1.234)			38.37 (8.228)			
	≥30,000	59 (5.76)	3.39 (1.474)			35.14 (9.096)			
**Region**					.03				.003
	Riyadh Region	150 (14.65)	3.67 (1.54)			37.95 (8.787)			
	Makkah Region	279 (27.25)	3.58 (1.43)			38.42 (7.641)			
	Eastern Region	111 (10.84)	3.59 (1.436)			37.53 (9.627)			
	Asir Region	63 (6.15)	3.44 (1.543)			36.35 (7.414)			
	Jizan Region	75 (7.32)	3.36 (1.43)			35.41 (9.015)			
	Almadinah Region	72 (7.03)	3.43 (1.412)			36.61 (8.564)			
	Alqassim Region	57 (5.57)	3.82 (1.351)			36.16 (7.615)			
	Tabuk Region	38 (3.71)	3.45 (1.501)			36.03 (8.952)			
	Haíl Region	36 (3.51)	3.03 (1.781)			35.97 (8.968)			
	Najran Region	28 (2.73)	3 (1.414)			31.93 (5.235)			
	Aljouf Region	30 (2.93)	3.67 (1.605)			37.37 (9.186)			
	Albaha Region	47 (4.59)	3.79 (1.382)			37.74 (9.073)			
	Northern Borders Region	38 (3.71)	2.84 (1.701)			34 (9.927)			
**Live in rural area**					<.001				<.001
	No	756 (73.83)	3.69 (1.426)			38.2 (8.353)			
	Yes	268 (26.17)	3.03 (1.548)			33.92 (8.224)			
**Have a chronic disease**					.78				.26
	No	610 (59.57)	3.53 (1.463)			37.33 (8.375)			
	Yes	414 (40.43)	3.5 (1.521)			36.71 (8.741)			
**Availability of car**					<.001				<.001
	No	290 (28.32)	3.06 (1.527)			34.52 (8.779)			
	Yes	734 (71.68)	3.7 (1.431)			38.09 (8.213)			
**Availability of smartphone**					<.001				<.001
	No	165 (16.11)	2.51 (1.213)			30.59 (6.681)			
	Yes	859 (83.89)	3.71 (1.456)			38.32 (8.276)			
**Availability of social media account**					<.001				<.001
	No	222 (21.68)	2.55 (1.36)			31.55 (7.714)			
	Yes	802 (78.32)	3.78 (1.408)			38.61 (8.1)			

^a^A 2-tailed Student *t* test was used for analysis of means of 2 groups, whereas 1-way ANOVA test was used for analysis of means of ≥3 groups.

^b^*P* value <.05 established statistical significance.

^c^SAR: Saudi riyal. SAR 1=US $0.27.

### Summary of the Surveyed Participants’ Knowledge of Telemedicine Services

Table 3 summarizes the participants’ knowledge of telemedicine. Overall, approximately three-quarters (738/1024, 72.07%) of the individuals had ever heard about telemedicine, but only 63.87% (654/1024) had actually seen a telemedicine process previously. The vast majority of participants reported that telemedicine reduced visit time (791/1024, 77.25%) and transportation costs (798/1024, 77.93%). Nonetheless, only 60.45% (619/1024) of the individuals declared that they had used telemedicine services before. There was a statistical difference between men and women regarding the following question: “does telemedicine reduce transportation costs?”; 75% (374/504) of the men answered “yes” compared with 81.5% (424/520) of the women (*P*=.005). In addition, individuals living in rural areas had statistically significant differences in several questions (n=4 questions; *P*=.01 to <.001) compared with individuals not living in rural areas. Overall, individuals living in rural areas had less knowledge of telemedicine services compared with individuals not living in rural areas.

**Table 3:** Surveyed participants’ knowledge of telemedicine (N=1024).

			All participants, n (%)		Gender analysis								Rural analysis						
					Men (n=504), n (%)		Women (n=520), n (%)		*P* value			No (n=756), n (%)				Yes (n=268), n (%)		*P* value	
**Have you heard the term telemedicine before?**										.78									<.001
	No	286 (27.9)		143 (28.4)		143 (27.5)					184 (24.3)				102 (38.1)				
	Yes	738 (72.1)		361 (71.6)		377 (72.5)					572 (75.7)				166 (61.9)				
**Have you seen a telemedicine process previously?**										.47									.01
	No	370 (36.1)		188 (37.3)		182 (35)					256 (33.9)				114 (42.5)				
	Yes	654 (63.9)		316 (62.7)		338 (65)					500 (66.1)				154 (57.5)				
**Does telemedicine reduce visit time?**										.10									<.001
	No	233 (22.8)		126 (25)		107 (20.6)					138 (18.3)				95 (35.4)				
	Yes	791 (77.3)		378 (75)		413 (79.4)					618 (81.7)				173 (64.6)				
**Does telemedicine reduce transportation costs?**										.005									<.001
	No	226 (22.1)		130 (25.8)		96 (18.5)					125 (16.5)				101 (37.7)				
	Yes	798 (77.9)		374 (74.2)		424 (81.5)					631 (83.5)				167 (62.3)				
**Have you ever used telemedicine services before?**										.16									.15
	No	405 (39.6)		188 (37.3)		217 (41.7)					289 (38.2)				116 (43.3)				
	Yes	619 (60.5)		316 (62.7)		303 (58.3)					467 (61.8)				152 (56.7)				

### Summary of the Participants’ Perceived Attitudes Toward Adoption of Telemedicine

Table 4 summarizes the perceived attitudes of the participants toward the adoption of telemedicine services. Overall, the statements with >50% of the surveyed individuals collectively reporting agree and strongly agree responses included “telemedicine is an effective tool for providing patient care” (631/1024, 61.6%), “telemedicine is cost-effective” (628/1024, 61.3%), “telemedicine can reduce unnecessary outpatient visits” (668/1024, 65.23%), and “telemedicine can be used to monitor chronic patients from home” (628/1024, 61.32%). Notably, only 41.89% (429/1024) and 41.41% (424/1024) of the individuals reported that “the legal aspects of telemedicine practice are clear” and “telemedicine threatens information privacy,” respectively.

The surveyed participants’ perceived attitudes toward the adoption of telemedicine services according to gender and rural area are summarized in Table 5. There was a statistical difference between men and women regarding the following attitude statement: “telemedicine can reduce unnecessary outpatient visits” (mean of men vs women: 3.53 vs 3.74; *P*=.005). In addition, individuals living in rural areas had statistically significant differences in several attitude statements (n=9 questions) compared with individuals not living in rural areas. Overall, individuals living in rural areas had fewer positive attitudes toward telemedicine services than the individuals not living in rural areas.

**Table 4:** Surveyed participants’ perceived attitudes toward adoption of telemedicine services.

	All participants (N=1024)					
	Strongly disagree, n (%)	Disagree, n (%)	Neutral, n (%)	Agree, n (%)	Strongly agree, n (%)	Value, mean (SD)
Telemedicine is an effective tool for providing patient care	108 (10.55)	96 (9.37)	189 (18.46)	470 (45.9)	161 (15.72)	3.5 (1.2)
Patients are satisfied with web-based consultations	103 (10.06)	148 (14.45)	292 (28.51)	368 (35.93)	113 (11.03)	3.2 (1.1)
The legal aspects of telemedicine practice are clear	99 (9.67)	193 (18.85)	303 (29.59)	318 (31.05)	111 (10.84)	3.2 (1.1)
Telemedicine is cost-effective	73 (7.13)	119 (11.62)	204 (19.92)	418 (40.82)	210 (20.51)	3.6 (1.1)
There is good scientific evidence for the use of telemedicine	72 (7.03)	120 (11.72)	331 (32.32)	376 (36.72)	125 (12.21)	3.4 (1.1)
Telemedicine shows good diagnostic concordance as face-to-face care	114 (11.13)	201 (19.63)	252 (24.61)	346 (33.79)	111 (10.84)	3.1 (1.2)
Telemedicine can reduce unnecessary outpatient visits	68 (6.64)	124 (12.11)	164 (16.01)	424 (41.41)	244 (23.83)	3.7 (1.2)
Effectiveness of telemedicine depends on the specialty	86 (8.4)	140 (13.67)	205 (20.02)	391 (38.18)	202 (19.73)	3.5 (1.2)
Telemedicine can be used to monitor chronic patients from home	74 (7.23)	116 (11.33)	207 (20.21)	396 (38.67)	231 (22.56)	3.6 (1.2)
Telemedicine increases communication	79 (7.71)	126 (12.3)	252 (24.61)	412 (40.23)	155 (15.14)	3.4 (1.1)
Telemedicine does not threaten information privacy	129 (12.6)	212 (20.7)	259 (25.29)	313 (30.57)	111 (10.84)	3.1 (1.2)

**Table 5:** Surveyed participants’ perceived attitudes toward adoption of telemedicine services according to gender and rural area.

	Gender analysis (men vs women)				Rural analysis (no vs yes)		
	Men (n=504), mean (SD)	Women (n=520), mean (SD)	*P* value	No (n=756), mean (SD)		Yes (n=268), mean (SD)	*P* value
Telemedicine is an effective tool for providing patient care	3.47 (1.217)	3.47 (1.137)	.99	3.60 (1.093)		3.11 (1.324)	<.001
Patients are satisfied with web-based consultations	3.24 (1.147)	3.23 (1.130)	.92	3.37 (1.324)		2.86 (1.236)	<.001
The legal aspects of telemedicine practice are clear	3.13 (1.131)	3.16 (1.149)	.61	3.19 (1.071)		3.01 (1.125)	.02
Telemedicine is cost-effective	3.53 (1.145)	3.58 (1.152)	.48	3.73 (1.236)		3.07 (1.231)	<.001
There is good scientific evidence for the use of telemedicine	3.36 (1.098)	3.35 (1.029)	.82	3.44 (1.142)		3.10 (1.110)	<.001
Telemedicine shows good diagnostic concordance as face-to-face care	3.19 (1.199)	3.08 (1.163)	.12	3.15 (1.125)		3.08 (1.187)	.39
Telemedicine can reduce unnecessary outpatient visits	3.53 (1.188)	3.74 (1.128)	.005	3.77 (1.065)		3.25 (1.212)	<.001
Effectiveness of telemedicine depends on the specialty	3.44 (1.223)	3.51 (1.163)	.35	3.59 (1.231)		3.13 (1.219)	<.001
Telemedicine can be used to monitor chronic patients from home	3.59 (1.144)	3.57 (1.185)	.80	3.73 (1.032)		3.17 (1.217)	<.001
Telemedicine increases communication	3.40 (1.122)	3.45 (1.122)	.45	3.58 (1.110)		2.99 (1.215)	<.001
Telemedicine does not threaten information privacy	3.08 (1.210)	3.05 (1.197)	.68	3.03 (1.180)		3.15 (1.204)	.19

### Summary of the Participants’ Perceived Barriers Toward Adoption of Telemedicine

Table 6 summarizes the perceived barriers of the participants toward the adoption of telemedicine services. Overall, no statements had >50% of the surveyed individuals collectively reporting agree and strongly agree responses. Interestingly, 45.31% (464/1024), 43.55% (446/1024), and 39.65% (406/1024) of the participants voiced that “physician resistance is a barrier to the practice of telemedicine,” “patient resistance is a barrier to the practice of telemedicine,” and “diagnostic reliability is a barrier to the practice of telemedicine,” respectively. Limitations to the adoption of telemedicine pertaining to culture and technology were voiced by 37.3% (382/1024) and 42.87% (439/1024) of the participants, respectively. Only 36.04% (369/1024) of the participants predicted that the “use of telemedicine will decrease after COVID-19 pandemic is over.”

**Table 6:** Surveyed participants’ perceived barriers toward adoption of telemedicine services.

	All participants (N=1024)					
	Strongly disagree, n (%)	Disagree, n (%)	Neutral, n (%)	Agree, n (%)	Strongly agree, n (%)	Value, mean (SD)
Physician resistance is a barrier to the practice of telemedicine	108 (10.54)	152 (14.84)	300 (29.31)	349 (34.08)	115 (11.23)	3.2 (1.2)
Patient resistance is a barrier to the practice of telemedicine	103 (10.06)	160 (15.62)	315 (30.76)	350 (34.18)	96 (9.38)	3.2 (1.1)
Diagnostic reliability is a barrier to the practice of telemedicine	91 (8.89)	205 (20.02)	322 (31.45)	318 (31.05)	88 (8.59)	3.1 (1.1)
Cultural aspects are a barrier to the practice of telemedicine	101 (9.86)	215 (21)	326 (31.84)	303 (29.59)	79 (7.71)	3 (1.1)
Technological limitations are a barrier to the practice of telemedicine	137 (13.38)	194 (18.95)	254 (24.8)	325 (31.74)	114 (11.13)	3.1 (1.2)
Use of telemedicine will decrease after the COVID-19 pandemic is over	180 (17.58)	209 (20.41)	266 (25.98)	283 (27.64)	86 (8.39)	2.9 (1.2)

The perceived barriers of the surveyed participants toward the adoption of telemedicine services according to gender and rural area are summarized in Table 7. There was no statistical difference in the mean scores between men and women for all statements. Nonetheless, individuals living in rural areas had statistically significant differences in 2 barrier statements (ie, patient resistance and diagnostic reliability; both *P*<.001) compared with individuals not living in rural areas. Overall, individuals living in rural areas had less barriers toward telemedicine services compared with individuals not living in rural areas.

**Table 7:** Surveyed participants’ perceived barriers toward adoption of telemedicine services according to gender and rural area.

	Gender analysis (men vs women)				Rural analysis (no vs yes)		
	Men (n=504), mean (SD)	Women (n=520), mean (SD)	*P* value	No (n=756), mean (SD)		Yes (n=268), mean (SD)	*P* value
Physician resistance is a barrier to the practice of telemedicine	3.22 (1.192)	3.19 (1.106)	.69	3.26 (1.092)		3.06 (1.287)	.18
Patient resistance is a barrier to the practice of telemedicine	3.14 (1.144)	3.20 (1.090)	.35	3.26 (1.069)		2.93 (1.209)	<.001
Diagnostic reliability is a barrier to the practice of telemedicine	3.08 (1.131)	3.13 (1.060)	.44	3.19 (1.079)		2.86 (1.108)	<.001
Cultural aspects are a barrier to the practice of telemedicine	3.09 (1.121)	2.99 (1.076)	.15	3.07 (1.077)		2.97 (1.159)	.18
Technological limitations are a barrier to the practice of telemedicine	3.04 (1.253)	3.13 (1.181)	.22	3.13 (1.222)		2.94 (1.193)	.02
Use of telemedicine will decrease after the COVID-19 pandemic is over	2.90 (1.221)	2.88 (1.236)	.72	2.87 (1.237)		2.94 (1.203)	.39

### Summary of the Cronbach α for Internal Consistency

The results for internal consistency showed an acceptable level of reliability for the attitudes survey (α=.878) and barriers survey (α=.781).

### Summary of the Multivariable Regression Analysis

Table 8 shows a summary of the multivariable regression analysis to identify sociodemographic variables related to knowledge and attitudes toward the adoption of telemedicine services. Increased scores imply increased knowledge and attitudes. Our results showed that there were positive and negative associations between various sociodemographic factors and the perceived knowledge and attitudes toward the adoption of telemedicine services. The findings revealed that gender had no impact on the scores of knowledge and attitudes. Conversely, married participants had significantly higher knowledge (β=.0222; *P*<.01) and positive attitudes (β=.0303; *P*<.01) than the participants in the reference group (nonmarried). Similarly, participants working in government-related jobs had significantly higher knowledge (β=.0262; *P*<.01) and positive attitudes (β=.0303; *P*<.05) than the participants in the reference group (unemployed). Salary had no impact on the attitude scores, whereas higher salary (>SAR 5000 to SAR 30,000) was significantly correlated with higher knowledge scores (*P*<.001). Moreover, advanced education (completion of high school and greater) was significantly linked to higher scores of knowledge and attitudes compared with the participants in the reference group (uneducated or primary school completed). For region, participants from Northern Borders Region had significantly lower knowledge scores (β=−.0440; *P*<.01) and less positive attitudes (β=−.0508; *P*<.05) than participants from the reference region (Riyadh). Saudi citizens had higher scores of knowledge (β=.0414; *P*<.01) and positive attitudes (β=.0566; *P*<.01) than the reference (non-Saudi) citizens. The availability of car, smartphone, and social media account positively correlated with higher scores of knowledge and positive attitudes compared with the reference groups that lacked the availability of such materials. Finally, living in rural area was negatively associated with significantly lower knowledge scores (β=−.0353; *P*<.01) and lesser positive attitudes (β=−.0521; *P*<.01) compared with participants not living in rural area (reference group).

**Table 8:** Multivariable regression analysis to identify sociodemographic variables related to knowledge and attitudes toward telemedicine services.

Variable			Knowledge, β (SE)	Attitudes, β (SE)
Gender (men)			−.0063 (.0051)	−.0024 (.0070)
**Age group (years)**				
	18-29	Reference group		Reference group
	30-39	.0133 (.0073)		.0108 (.0100)
	40-49	−.0034 (.0078)		−.0226 (.0107)^a^
	50-59	−.0084 (.0084)		−.0062 (.0116)
	≥60	−.0059 (.0096)		−.0194 (.0132)
**Marital status**				
	Nonmarried	Reference group		Reference group
	Married	.0222 (.0051)^b^		.0303 (.0070)^b^
**Job**				
	Unemployed	Reference group		Reference group
	Governmental	.0262 (.0084)^b^		.0265 (.0116)^a^
	Nongovernmental	.0150 (.0091)		.0056 (.0125)
	Self-employed	−.0203 (.0104)		−.0437 (.0143)^b^
	Student	−.0095 (.0101)		−.0230 (.0139)
	Retired	−.0042 (.103)		−.0144 (.0142)
**Salary (SAR^c^)**				
	<3000	Reference group		Reference group
	3000 to <5000	.0103 (.0103)		.0041 (.0144)
	5000 to <7000	.0308 (.0095)^b^		.0125 (.0132)
	7000 to <10,000	.0360 (.0093)^b^		.0097 (.0130)
	10,000 to <15,000	.0327 (.0087)^b^		.0151 (.0121)
	15,000 to <20,000	.0338 (.0094)^b^		.0255 (.0131)
	20,000 to <30,000	.0463 (.0105)^b^		.0279 (.0146)
	≥30,000	.0202 (.0124)		−.0139 (.0172)
**Region**				
	Riyadh Region	Reference group		Reference group
	Makkah	−.0035 (.0082)		.0102 (.0113)
	Eastern Region	−.0023 (.0102)		−.0068 (.0140)
	Asir Region	−.0110 (.0122)		−.0135 (.0168)
	Jizan Region	−.0142 (.0115)		−.0299 (.0158)
	Almadinah Region	−.0100 (.0117)		−.0156 (.0160)
	Alqassim Region	.0107 (.0127)		−.0160 (.0174)
	Tabuk Region	−.0101 (.0148)		−.0206 (.0203)
	Haíl Region	−.0351 (.0151)^a^		−.0226 (.0208)
	Najran Region	−.0325 (.0168)		−.0643 (.0230)^b^
	Aljouf Region	.0004 (.0163)		−.0052 (.0224)
	Albaha Region	.0075 (.0136)		−.0015 (.0187)
	Northern Borders Region	−.0440 (.0148)^b^		−.0508 (.0203)^a^
**Education**				
	Primary school completed and below	Reference group		Reference group
	Intermediate school completed	.0131 (.0110)		.0257 (.0152)
	High school completed	.0583 (.0092)^b^		.0730 (.0127)^b^
	Diploma before university	.0529 (.0098)^b^		.0628 (.0135)^b^
	College or university degree completed	.0611 (.0084)^b^		.0872 (.0115)^b^
	Postgraduate degree completed	.0609 (.0092)^b^		.0786 (.0127)^b^
**Nationality**				
	Non-Saudi	Reference group		Reference group
	Saudi	.0414 (.0058)^b^		.0566 (.0080)^b^
Live in rural area (yes)			−.0353 (.0057)^b^	−.0521 (.0078)^b^
Presence of chronic disease (yes)			−.0012 (.0052)	−.0072 (.0072)
Availability of car (yes)			.0345 (.0056)^b^	.0465 (.0077)^b^
Availability of smartphone (yes)			.0625 (.0067)^b^	.0965 (.0091)^b^
Availability of social media account (yes)			.0660 (.0059)^b^	.0899 (.0081)^b^
**Family members**				
	≤5	Reference group		Reference group
	≥6	−.0106 (.0055)		−.0026 (.0076)

^a^Statistical significance with *P*<.05.

^b^Statistical significance with *P*<.001.

^c^SAR: Saudi riyal. SAR 1=US $0.27.

## Discussion

### Principal Findings

This study explored the knowledge and the perceived attitudes and barriers toward the application of telemedicine services in the Kingdom of Saudi Arabia. Overall, 1024 participants took part in the survey, and the demographics were largely balanced. Overall, 72.07% (738/1024) of the participants reported that they had heard about telemedicine, but only 63.87% (654/1024) of the participants had seen a telemedicine process previously. The perceived attitudes were moderate, whereby approximately 40% to 60% of the participants expressed positive attitudes toward the benefits of telemedicine services. As for the perceived barriers, the participants voiced some concerns regarding patient and physician resistance and attributed some cultural and technology-related limitations as potential hindrances to the full adoption of telemedicine services. There were no significant differences in the knowledge and the perceived attitudes and barriers toward the application of telemedicine services in the Kingdom of Saudi Arabia between men and women. Nonetheless, the place of residence (rural vs nonrural) had a significant impact on the knowledge and perceived attitudes and barriers toward the application of telemedicine services in the Kingdom of Saudi Arabia. Finally, there were significant positive and negative associations between various sociodemographic factors and the perceived knowledge and attitudes toward the adoption of telemedicine services.

### Research Contributions

Considering the mismatch between the presence of telemedicine programs in the Kingdom of Saudi Arabia [6,21] and poor acceptance by end users [13], particularly patients, there is an urgent necessity to gain deeper insights into the elements that impact the progress of telemedicine adoption in the Kingdom of Saudi Arabia. Unfortunately, the current body of literature is poor with respect to data regarding the perceptions, attitudes, and knowledge of the end-user patients toward the utility and barriers of telemedicine. Without many insights about the abovementioned factors and contributors, policy makers will not be able to positively enforce the strengths and positive attitudes and resolve the related barriers. This research advantageously kicks in to fill out this literature gap by diving into the adoption of telemedicine services by participants, its determinants of utility, and its perceived obstacles.

The goal of undertaking this research was to generate valuable data that can be exploited by health care stakeholders to inform strategic and focused directions to maximize the utility of telemedicine services. Maximization of such telemedicine services is increasingly becoming more important, particularly nowadays amid the wide-ranging emergence of global outbreaks, such as the COVID-19 pandemic [22,23]. Finally, this study will enrich the body of local or regional literature on the adoption of telemedicine among participants in the Kingdom of Saudi Arabia. The findings from this research will provide valuable material for high-quality systematic reviews and meta-analyses, contributing to the generation of concrete conclusions.

### Interpretation of Findings and Comparison With Prior Work

A key aspect of any survey is to examine its reliability. Cronbach α is a well-established metric to gauge the extent of internal consistency, by measuring how closely a related set of items come together as a group. It is largely regarded as a metric of scale reliability [19]. Our results showed that both questionnaires on perceived attitudes and barriers were highly reliable, as reflected by the α values of >.70. Validity is another instrumental metric to be examined in any survey [24].

The available literature is very anemic regarding the perceptions of patients toward the adoption of telemedicine services in the Kingdom of Saudi Arabia [13], and this constituted our primary drive behind conducting this research. We conducted this research at a nationwide scale to produce a largely countrywide and well-represented landscape of the telemedicine services in the Kingdom of Saudi Arabia. Most importantly, the gender distribution was well balanced in terms of proportions, and gender was used to examine differential changes in the knowledge and the perceived attitudes and barriers toward the application of telemedicine services in the Kingdom of Saudi Arabia between men and women. The Kingdom of Saudi Arabia is a largely very “conservative” society when compared with other Islamic countries. Indeed, the lack of adequate knowledge on the influence of gender-related aspects of telemedicine use among individuals in Saudi Arabia has been recently highlighted as a recommended area for future research [14].

In our study, it was very disappointing to note that the proportions of individuals who attended a telemedicine service before, during, and after COVID-19 oscillated between 50% and 60%. These proportions were far below the desired targets. Therefore, considering the mismatch between the presence of telemedicine programs in the Kingdom of Saudi Arabia [6,21] and poor acceptance by the end users, particularly patients [13], there is an urgent need to gain deeper insights into the elements that impact the progress of telemedicine adoption in the Kingdom of Saudi Arabia.

The perceived attitudes of participants toward the benefits of telemedicine services were moderate. In contrast, the reported perceived barriers toward the adoption of telemedicine services were largely in line with the published literature both regionally [10,13,17,21,25] and internationally [26,27]. As it can be observed from the timeline of the telemedicine progress (Figure 1), it can be stated that telemedicine has experienced vivid modifications in the past decade in response to the technological advances in the country [6]. Nevertheless, a multitude of barriers have been chronicled with respect to the application of telemedicine programs in the Kingdom of Saudi Arabia. The major barriers toward the full adoption of telemedicine services in the Kingdom of Saudi Arabia can be broadly categorized into 6 categories. These categories comprise technological, organizational, financial, legal, cultural, and individual barriers. The barriers that mostly concern patients include the legal, cultural, and individual barriers. Nonetheless, the technological, organizational, and financial barriers can also affect the patients directly or indirectly [10,11,13,17,21,25].

Notably, our study showed that participants expressed an overall neutral response to cultural aspects as potential barriers to telemedicine practice. Cultural barriers pertaining to efficient use of telemedicine programs are largely affected by social restrictions, religious beliefs, literacy, resistance to change, and linguistic obstacles. Similar to other Middle Eastern countries, in the Kingdom of Saudi Arabia, the acceptance of a new health care system is dependent on a variety of religious, social, and cultural barriers. Indeed, social and religious restrictions are ranked first as the top challenges in accepting telemedicine services. The culture of the Kingdom of Saudi Arabia, to a larger degree, is very conservative, particularly with respect to the interaction between both genders (men and women). A growing pool of literature highlights the apprehension of women (whether physicians or patients) to be recorded during telemedicine encounters. There are ongoing fears that such filmed encounters may be disclosed to unauthorized personnel or shared publicly in media without approval. It is naturally expected that adaptation to a new system will be challenging, and this is also true and applicable to the adoption of telemedicine by both physicians and patients. Thus, resistance to change is a major problem encountered during the implementation of telemedicine programs. In addition, it must be noted that illiteracy is a pivotal cultural element that can severely impact the utility of telemedicine services. Language barrier is also a crucial element that can modulate the success or failure of physician-patient encounters during telemedicine services. Arabic is the standard language in the Kingdom of Saudi Arabia, and many foreign physicians are unable to communicate in Arabic. Unfortunately, in our survey, we did not precisely articulate these specific cultural aspects, and these can be addressed in depth in future research [6,11,13,25].

### Strengths and Limitations

To the best of our knowledge, this is the largest, nationwide, and survey-based investigation on the knowledge and perceived attitudes and barriers toward telemedicine services in the Kingdom of Saudi Arabia. This study is important conceptually and methodologically. From a conceptual perspective, this research focused on telemedicine, which is a very important topic relevant to health care. This importance of telemedicine is demonstrated by the wide array of benefits that are largely stated in the literature [7]. From a methodological perspective, this study used a quantitative approach to draw high-quality and concrete conclusions [28]. Our survey was devised based on extensive literature review and subjected to a stringent examination of validity and reliability to reduce the potential of analytical and reporting bias. Other strengths include the large sample size of the surveyed participants, which adds power to the deduced results.

Nonetheless, this study is not without limitations, and these shortcomings should be acknowledged. Most importantly, as this study was self-reported, the results could be liable to underestimation or overestimation by the surveyed participants. Moreover, it was not technically possible to verify the statements of the participants. Moreover, as this study was cross-sectional, it would not be possible to examine the changes in the surveyed participants’ knowledge, attitudes, and barriers over time. In addition, causal relationships could not be established. In addition, several confounding factors (eg, nationality, presence of social media account, and residence in rural area) could have indirectly affected the pooled outcomes. Although this study was randomized and anonymous, a selection bias could not be excluded. Finally, given the wide distribution of the survey via various social media platforms, it was not possible to determine the response rate.

### Conclusions

This nationwide investigation explored the knowledge and the perceived attitudes and barriers toward the application of telemedicine services in the Kingdom of Saudi Arabia among 1024 research participants. The perceived attitudes were moderate whereby approximately 40% to 60% of the participants expressed positive attitudes toward the benefits of telemedicine services. The perceived barriers were in line with the published literature and included some concerns regarding patient and physician resistance and some cultural and technology-related limitations as potential hindrances to the full adoption of telemedicine services.

In terms of implications, this research highlights the need to adopt a standard definition of telemedicine services so that all stakeholders (patients, physicians, and governing bodies) can be on the same page. Finally, there is a need for more holistic research and funding to better boost the quality and minimize the costs of telemedicine services.

In view of the small number of related studies on the subject, additional large-scale, survey-based investigations are warranted to consolidate the findings. In addition, to further ensure that the outcomes of this research will be translated into tangible benefits, an advisory collaboration with the Ministry of Health officials should be sought. This research calls for serious efforts to develop focused and informed strategies to strengthen positive attitudes toward telemedicine services and to devise practical short- and long-term schemes to rectify the barriers toward the adoption of telemedicine services by participants in the Kingdom of Saudi Arabia.

## Abbreviations

SAR: Saudi riyal
